# Contrasting Patterns of Inbreeding and Inbreeding Depression in Co‐Occurring Spotted Turtle and Eastern Box Turtle Populations

**DOI:** 10.1002/ece3.72736

**Published:** 2025-12-17

**Authors:** Paige K. Madden, Sarah E. Carter, Austin C. Hulbert, Henry M. Streby, Jeanine M. Refsnider

**Affiliations:** ^1^ Department of Environmental Sciences University of Toledo Toledo Ohio USA

**Keywords:** *Clemmys guttata*, heterophil:lymphocyte ratio, home range, radio‐telemetry, reproductive success, *Terrapene carolina*

## Abstract

Maintenance of genetic diversity is critical for preserving a population's ability to adapt to changing selection pressures. As populations become smaller and more isolated, they are more likely to become inbred, which can lead to inbreeding depression and further population decline. Eastern box turtles and spotted turtles co‐occur in remnant oak savanna landscapes in Ohio and Michigan, and while both species have experienced population decline due to habitat loss, remaining spotted turtle populations are smaller and more isolated than box turtle populations; thus, spotted turtles may be more vulnerable to inbreeding depression than eastern box turtles. To test this prediction, we estimated population size, measured the total area occupied, home range size, and baseline physiological stress levels in three populations of both eastern box turtles and spotted turtles. We then compared the inbreeding coefficient and two metrics of reproductive success (egg‐hatching success and hatchling survival to overwintering) as potential evidence of inbreeding depression between the two species. We found that while spotted turtles had smaller population sizes, occupied smaller habitat patches, had smaller home ranges, and had higher baseline physiological stress levels than box turtles, the box turtle populations had higher inbreeding coefficients. Moreover, lower rates of egg‐hatching success and hatchling survival in the box turtle compared to the spotted turtle populations suggest that the box turtle populations may be experiencing inbreeding depression, whereas inbreeding depression was not evident in the spotted turtle populations. Habitat loss and fragmentation may have contributed to inbreeding effects in box turtles by obstructing dispersal that would otherwise promote mating among unrelated conspecifics. A top priority for conserving these vulnerable turtles should be the preservation of intact tracts of habitat, restoring connectivity among sub‐populations where possible, and preventing further fragmentation of occupied habitat by roads in order to promote gene flow and random mating.

## Graphical abstract

We found that while spotted turtles had smaller population sizes, occupied smaller habitat patches, had smaller home ranges, and had higher baseline physiological stress levels than box turtles, the box turtle populations had much higher inbreeding coefficients than the spotted turtle populations. Moreover, lower rates of egg‐hatching success and hatchling survival to overwintering in the eastern box turtle compared to the spotted turtle populations suggest that the box turtle populations may be experiencing inbreeding depression, whereas inbreeding depression was not evident in the spotted turtle populations, all of which demonstrated high rates of both egg‐hatching success and hatchling survival to overwintering.

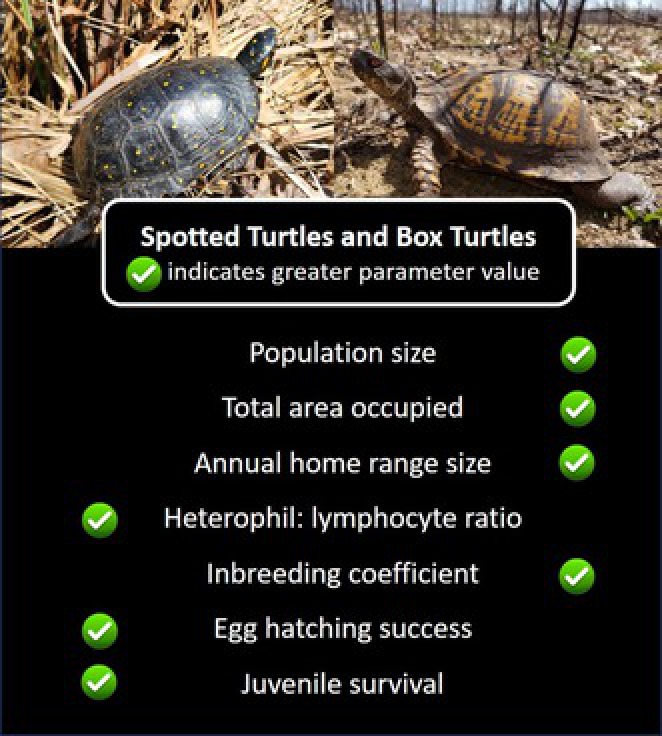

## 1 Introduction

Genetic diversity is critical for population persistence because it provides the raw genetic material coding for phenotypes on which natural selection can act. Thus, a population's genetic diversity is directly related to its potential to adapt to changing environmental pressures (Frankham 2005). As population size decreases, genetic diversity also generally decreases, such that small populations become especially vulnerable to non‐adaptive processes such as genetic drift and inbreeding depression, and are more likely to lose their evolutionary potential to adapt to a changing environment (Lande 1995; Falconer and Mackay 1996). In small populations with a high frequency of inbreeding, offspring of matings between close relatives are more likely to be homozygous for deleterious recessive alleles than are offspring of unrelated parents (Keller and Waller 2002). Deleterious traits expressed by inbred offspring that result in lower survival or reproductive viability compared to outbred offspring are evidence that a population is experiencing inbreeding depression (e.g., Hedrick and Kalinowski 2000; Billing et al. 2012; Willoughby et al. 2019; Duntsch et al. 2023). The cycle whereby small populations experience increasing inbreeding depression, which further decreases population size and erodes genetic diversity, becomes a feedback loop often termed an “extinction vortex” (Gilpin and Soulé 1986; Blomqvist et al. 2010).

Inbreeding tends to occur at higher frequency in small populations compared to large populations, in which random mating is statistically more probable (Hedrick and Kalinowski 2000; Billing et al. 2012). Inbreeding may also be more common in environments where individuals are spatially constrained, such as highly fragmented landscapes where populations occur in patches surrounded by inhospitable habitat that prevents individuals from dispersing and mating with unrelated conspecifics (e.g., Sambatti et al. 2008; Willoughby et al. 2019; Tian et al. 2022). Finally, the fitness consequences of inbreeding may be greater in more stressful environments compared to less stressful environments (Armbruster and Reed 2005; Fox and Reed 2010). Combined, these factors mean that threatened species—which are often characterized by small and isolated populations experiencing multiple environmental stressors—are particularly at risk of inbreeding and potentially inbreeding depression, which could further decrease population size and genetic diversity (Daniels and Walters 2000; Hoffmann et al. 2017).

Turtles are one of the world's most globally imperiled taxa, with 56% of extant species classified as Threatened by the IUCN (Rhodin et al. 2018). The long lifespan and cryptic nature of many turtle species means that recruitment failure and population declines may go unnoticed for many years before conservation actions are taken (Marsack and Swanson 2009). Even when population trends and levels of genetic diversity are known for a given species, the genetic structures of most turtle populations are more likely to reflect historical, rather than contemporary, processes (Davy and Murphy 2014); thus, the impacts of recent anthropogenic effects such as habitat fragmentation or climate change on the genetic diversity of turtle populations may not yet be detectable (Marsack and Swanson 2009). It is rarely feasible to compare genetic parameters such as allelic richness or rate of inbreeding for a turtle population over a sufficiently long timeframe to determine whether the population's genetic health has declined over several generations. However, in some situations it may be possible to compare population genetic parameters of co‐occurring turtle species to determine how genetic diversity might be impacted differently among species experiencing the same environmental pressures.

In the North American Great Lakes region, the glacial and lake sand plain was historically a landscape mosaic comprising oak savanna, grassland, and wet prairie habitat (Nuzzo 1986), which supported many unique taxa (Grigore 2009). The vast majority of this landscape was subsequently converted for agricultural use and extensively fragmented (Leach and Givnish 1999), such that remnant natural communities now occur in small, isolated patches. Two turtle species that co‐occur in the oak savanna landscape of the North American Great Lakes region are the spotted turtle ( *Clemmys guttata* ) and the eastern box turtle ( *Terrapene carolina carolina* ). Populations of both species have declined substantially across their geographic range due to habitat loss and over‐collection for the pet trade, and both species are now designated as threatened or endangered across much of their range (van Dijk 2011a, 2011b). Both spotted turtles and eastern box turtles are long‐lived, late‐maturing, and produce relatively small clutches (Ernst and Lovich 2009), meaning that population recovery following a decline is likely to be slow, if it occurs at all. Spotted turtles are more strongly associated with seasonally wet habitat compared to eastern box turtles, and at our study sites are mainly found in wet prairie, swamp forest, and fen habitat (Refsnider et al. 2022). In contrast, eastern box turtles use oak savanna and mixed hardwood forest habitat, with seasonal travel to open grassland habitat for nesting (Refsnider et al. 2022).

Due to their designation as species of high priority for conservation, both spotted turtles and eastern box turtles have been the focus of numerous studies on population trends, habitat use, spatial ecology, and impacts of management activities (e.g., Litzgus and Mousseau 2004; Enneson and Litzgus 2008; Melvin and Roloff 2018; Harris et al. 2020; O'Dell et al. 2021). Population estimates from such studies are generally lower for spotted turtles compared to eastern box turtles (Enneson and Litzgus 2009; Marsack and Swanson 2009; Buchanan et al. 2019; Moore et al. 2020). Similarly, home range sizes of individual spotted turtles are generally smaller than those of eastern box turtles (Buchanan et al. 2017; Habeck et al. 2019; Roe et al. 2020; O'Dell et al. 2021). Studies incorporating genetic relatedness demonstrate that eastern box turtles exhibit male‐biased dispersal patterns (Moore et al. 2020), whereas no sex‐biased dispersal was evident in spotted turtles (Liebgold et al. 2023). Despite often small population sizes and apparent lack of recent gene flow, spotted turtle populations generally do not exhibit significant inbreeding or evidence of declining genetic diversity (Davy and Murphy 2014; Anthonysamy et al. 2018; Buchanan et al. 2019). Eastern box turtle populations near the center of their geographic range show low levels of population structure but some evidence of a bottleneck (Marsack and Swanson 2009), but populations towards the northern range limit exhibit lower genetic diversity and more genetic clustering attributable to restricted female dispersal away from nesting areas (Moore et al. 2020). Overall, the combination of smaller population sizes, shorter distances traversed by individuals, and lack of sex‐biased dispersal in spotted turtles compared to eastern box turtles suggests that spotted turtle populations may be more vulnerable to inbreeding and inbreeding depression compared to populations of eastern box turtles.

We tested the prediction that spotted turtles have higher rates of inbreeding and exhibit evidence of inbreeding depression compared to eastern box turtles in the same region at three sites for each species; the two species co‐occurred at two of the study sites. For each study population of each species, we compared population size using mark‐recapture records, measured the total area occupied by the species, quantified individual home range size, and measured baseline physiological stress levels as a proxy for the “environmental stress” being experienced by each population. These measures served to test the general trend from other populations that spotted turtles have smaller population sizes, smaller home ranges, and are experiencing greater environmental stress than eastern box turtles. Next, we genotyped adults of each species at 6–8 microsatellite loci to quantify genetic diversity and frequency of inbreeding in each population. Finally, as potential evidence of inbreeding depression, we used two different metrics of fitness associated with early life stages, as strong inbreeding depression should most affect juveniles. We compared egg‐hatching success and survival of hatchlings to overwintering between the two species to determine if higher frequencies of inbreeding were associated with lower metrics of reproductive success in either spotted turtles or eastern box turtles.

## 2 Materials and Methods

### 2.1 Study Sites

**FIGURE 1:**
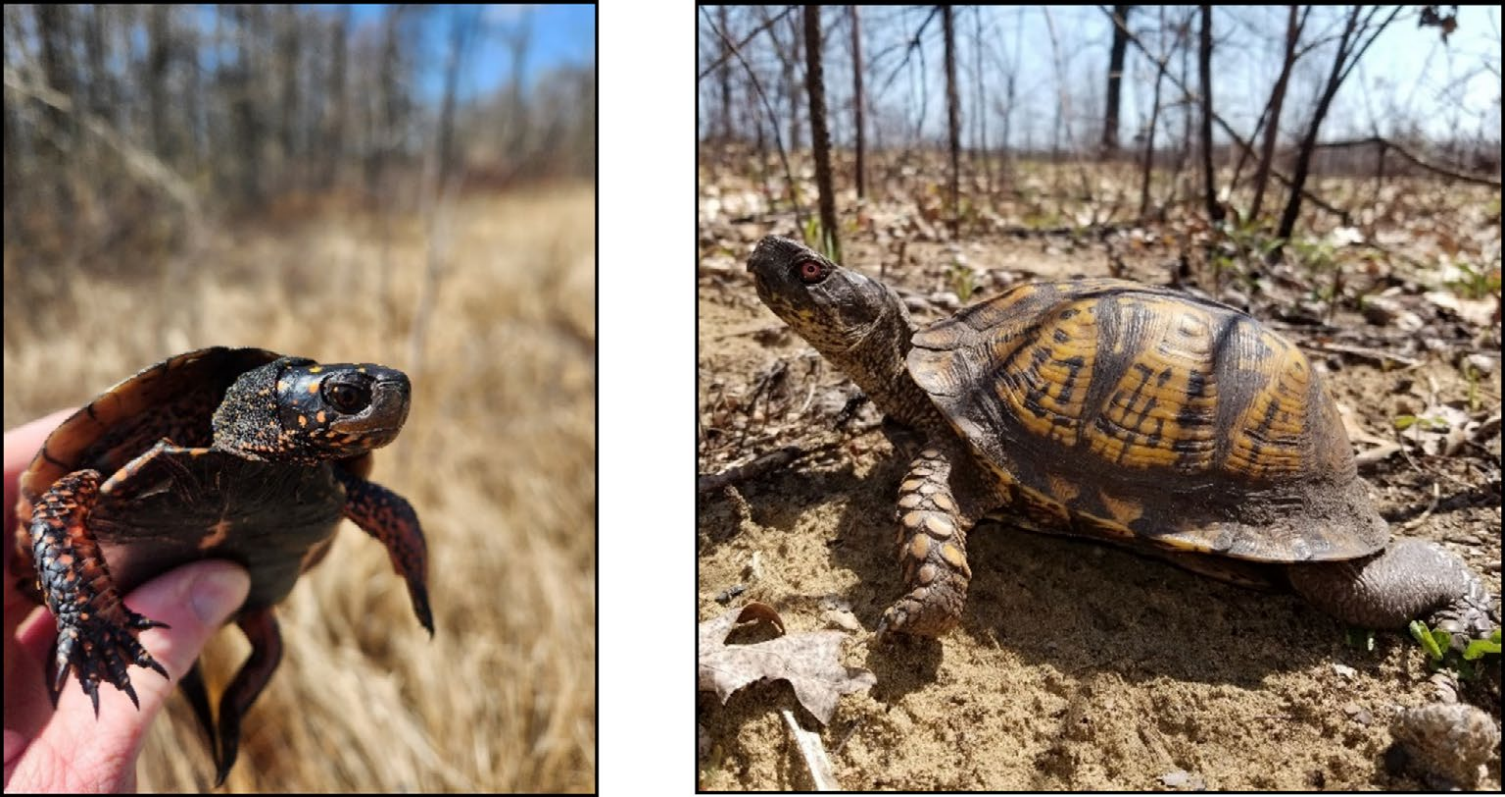
Spotted turtle ( *Clemmys guttata* , left) and eastern box turtle ( *Terrapene carolina carolina* ) in Ohio, USA. Photo credits: H. Streby and J. Refsnider.

We sampled spotted turtles and eastern box turtles (Figure 1) from three study populations of each species in 2018 and 2019 (Hulbert 2021; Carter 2021). Exact locality data are being withheld due to the sensitivity of these species to poaching. The two species co‐occur at Study Sites 1 and 2, which were in Lucas County, Ohio, USA. Only spotted turtles occurred at Site 3 (Barry County, Michigan, USA) and only eastern box turtles occurred at Site 4 (Calhoun County, Michigan, USA).

### 2.2 Population Size, Total Area Occupied, Home Range Sizes, and Baseline Stress

We conducted extensive visual encounter surveys for both species in early spring (April—May) in 2018 and 2019 at each study site. Adult females of both species were outfitted with radio‐transmitters to locate nest sites (ATS Inc. and Holohil Transmitters; see detailed methods in Refsnider et al. 2022), and during extensive radio‐telemetry activities in May and June we opportunistically encountered additional unmarked adult turtles of both species. Any time an adult turtle not outfitted with a radio‐transmitter was encountered in this study, we first determined if it had previously been marked by scanning it with a passive integrated transponder (PIT) reader and checking its carapace for shell notches. All adult turtles of both species that had not previously been marked were individually marked upon their initial capture in this study. In Ohio, in accordance with research permits issued by the Ohio Department of Natural Resources, turtles of both species were injected with individually numbered PIT tags. In Michigan, turtles of both species were marked by filing a unique combination of notches into the marginal scutes (as in Cagle 1939). We also recorded capture location of all individuals and radio‐telemetry locations of turtles being radio‐tracked, using a handheld GPS unit (Garmin; Refsnider et al. 2022). Finally, the first time we encountered an adult turtle of either species in this study, we collected a ~1 mL blood sample from the caudal vein using a heparinized, 28‐gauge needle. One drop of blood was used to make a blood smear on a glass slide to quantify baseline stress levels (see below). Turtles that were re‐captured after having previously been marked were identified by their PIT tag number or shell notch combination and released at their recapture location without additional sampling. We estimated population sizes for each species at each of the three sites using the Cormack–Jolly–Seber method, with 2018 and 2019 as the two unique sampling events, and individuals that were encountered at least once during both years included as “recaptures.”

We plotted all radio‐telemetry locations of radio‐tracked turtles, and the initial capture locations of all turtles, in a GIS (ArcMap, Esri). For each study population of both species, we created a minimum convex polygon (MCP) that encompassed the outermost points of all radio‐telemetry and initial capture locations for all adults encountered in each study population. We then used the area of each such MCP as an estimate of the total area occupied by the corresponding study population.

Individual home range sizes were calculated from radio‐telemetry data from adult female turtles of both species that were radio‐tracked over a full active season (i.e., April–October) in 2018 or 2019 (Carter 2021). Briefly, for female turtles that were radio‐tracked over the course of an entire active season, we calculated each individual's MCP for that active season using one radio‐location per day (Carter 2021). Thus, for females where we had two full seasons of radio‐telemetry locations, we calculated two separate annual MCPs, one for each year. Within each study population, we used the mean of all annual MCPs calculated for the radio‐tracked adult females as the mean annual home range size for that study population. We compared mean annual home range size between spotted turtles and box turtles using *t*‐tests.

We measured baseline physiological stress levels of each individual by quantifying the heterophil:lymphocyte (H:L) ratio from the blood smear collected upon each individual's initial capture. Blood smears were air dried in the field at the time of collection, subsequently fixed in 70% ethanol, and stained with Stat‐Quick Wright Giemsa and May–Grünwald Stain (ENG Scientific Inc.). To quantify each individual's H:L ratio, we counted 100 white blood cells in each blood smear at 1000× power using a light microscope and classified each white blood cell as a heterophil, lymphocyte, eosinophil, basophil, or monocyte (Kassab et al. 2009; Javanbakht et al. 2013). The ratio of heterophils to lymphocytes was used as each individual's baseline stress level (as in Refsnider, Garcia, et al. 2021), and the mean H:L ratio of each population served as an indicator of the “environmental stress” a given population was experiencing, such that higher H:L ratios indicate higher levels of baseline physiological stress (Davis et al. 2008). We first compared mean H:L ratios between males and females within species using *t*‐tests. As the sexes did not differ in mean H:L ratios for either spotted turtles (*p* = 0.80) or box turtles (*p* = 0.50), we then used a *t*‐test to compare overall mean H:L ratios between the two species.

### 2.3 Genetic Diversity and Inbreeding Coefficient

We extracted DNA from blood samples collected upon initial capture from adult spotted turtles and eastern box turtles using a 10% solution of Chelex resin (Bio‐Rad). We amplified spotted turtle DNA at eight microsatellite loci (*Gmu*D16, *Gmu*D21, *Gmu*D55, *Gmu*D70, *Gmu*D79, *Gmu*D87, *Gmu*D93, and *Gmu*D107) previously developed for bog turtles (King and Julian 2004), and we amplified eastern box turtle DNA at six microsatellite loci (TCC_di_045, TCC_di_082, TCC_di_300, TCC_di_318, TCC_di_345, and TCC_tetra_012/342) using species‐specific primers developed by Kimble et al. (2011). All microsatellites for both species were sized and visualized using AB GeneMapper software. For each study population of each species, we used the package Genepop (version 1.2.2; Raymond and Rousset 1995) in R (R Core Team 2022) to calculate observed and expected heterozygosity, and mean number of private alleles (i.e., unique alleles observed in only a single population; also see Madden 2023). We also used Genepop to calculate the inbreeding coefficient *F*_is_ for each study population, which is defined as the deviation of the observed heterozygosity of an individual relative to the heterozygosity expected under random mating in a population in Hardy–Weinberg equilibrium, or *F*_is_ = 1 − *H*_o_/*H*_e_ (Keller and Waller 2002). Thus, *F*_is_ > 0 signifies more inbreeding than would be expected under random mating (Keller and Waller 2002). Finally, to account for differences in sample sizes among the populations (particularly for box turtles; as in El Mousadik and Petit 1996), we used the R packages hierfstat (version 0.5‐11; Goudet 2005) and pegas (Paradis 2010) to calculate mean number of rarefied alleles for each population of each species as an indicator of genetic diversity.

### 2.4 Egg‐Hatching Success and Hatchling Survival to Overwintering

We used two metrics of reproductive success, egg‐hatching success and hatchling survival to overwintering, to compare between species for potential evidence of inbreeding depression. The dataset and estimates of egg‐hatching success and hatchling survival to overwintering for both species have been previously published (Refsnider et al. 2022; dataset available in Refsnider, Carter, et al. 2021, “SWGHatchlings.csv”). Briefly, upon initial capture in the spring of one or both years of the study, gravid females of both species were outfitted with radio‐transmitters and were extensively radio‐tracked during the May–June nesting season of 2018 and/or 2019 to locate nest sites (see detailed methods in Refsnider et al. 2022). Within 24 h of construction, we briefly excavated nests to record clutch size, before replacing the eggs in the nest chamber and re‐filling the nest with substrate. We protected most nests with wire cages that both prevented nest predation and served to contain hatchlings upon emergence (Refsnider et al. 2022). Beginning in early August, we monitored nest cages every 1–3 days for hatchling emergence. When hatchlings were observed within the nest cage, we excavated the rest of the nest to quantify egg‐hatching success, which was recorded as number of living hatchlings/initial clutch size. If no hatchlings had emerged within 100 days of nest construction, we excavated the nest to confirm that no hatchlings had been produced, and egg‐hatching success was recorded as 0 (Refsnider et al. 2022).

To quantify hatchling survival to overwintering, we attached miniature radio‐transmitters (Blackburn Transmitters) to two hatchlings per nest and radio‐tracked them 1–3 times per week until the signal was lost, mortality was confirmed, or the hatchling entered hibernation in October or November (see detailed methods in Hulbert 2021; Refsnider et al. 2022). We estimated hatchling survival to overwintering in two ways: first, we used a conservative estimate in which we only included hatchlings whose fates were known. For this conservative estimate, we classified hatchlings that were either found dead or whose transmitters were found with damage consistent with an attack by a predator as not surviving to overwintering; hatchlings that were known to have entered hibernation were classified as surviving to overwintering. As a secondary estimate of hatchling survival to overwintering, we included hatchlings whose fates were presumed but not known with certainty. For this less conservative estimate, hatchlings whose radio signals were lost more than 3 days before predicted transmitter battery expiration were assumed to have been depredated, and therefore had not survived to overwintering. Hatchlings whose radio signals were lost after 1 October and within 3 days of predicted battery expiration were presumed to have survived to overwintering, as most surviving hatchlings had reached their eventual overwintering location by 1 October. If a hatchling's radio signal was lost within 3 days of expected battery failure but the signal loss occurred before 1 October, we classified the hatchling's fate as unknown and excluded it from analysis.

## 3 Results

### 3.1 Population Size, Total Area Occupied, Home Range Sizes, and Baseline Physiological Stress

We found that the spotted turtle study populations were generally smaller, occupied a smaller area, had smaller mean home ranges, and had higher mean baseline stress levels than the eastern box turtle populations (Table 1). Population estimates for spotted turtles at each study site ranged from 30 to 50, whereas population estimates were 27–212 at the three box turtle study sites. The area occupied by the three study populations of spotted turtles ranged from 9 to 33 ha, compared to 175 to 930 ha occupied by the eastern box turtle study populations (Table 1). Adult female spotted turtles had mean annual home ranges of 2.88 ± 0.31 ha across the three study populations, whereas adult female box turtle mean annual home ranges were 7.52 ± 1.18 ha (*t* = −3.79, *p* = 0.0004; Table 1). Finally, mean baseline physiological stress levels, measured as H:L ratios, were 0.884 ± 0.06 across the three spotted turtle study populations, and 0.714 ± 0.05 across the three eastern box turtle study populations (*t* = 2.15, *p* = 0.033; Table 1).

**TABLE 1:** Estimated population size (Cormack–Jolly–Seber method), estimated total area occupied, mean annual female home range size (95% minimum convex polygon), and mean heterophil:lymphocyte (H:L) ratio in three study populations of spotted turtles ( *Clemmys guttata* ) and eastern box turtles ( *Terrapene carolina carolina* ) in Lucas County, Ohio (Pops 1 and 2), and Barry (Pop 3) and Calhoun Counties (Pop 4), Michigan, USA in 2018 and 2019.

	Population size	Total area occupied (ha)	Mean ± SD annual female home range size (ha)	Mean ± SD adult H:L
Spotted turtles				
Pop 1	30	33.36	3.68 ± 0.54 (*n* = 10)	0.822 ± 0.89 (*n* = 26)
Pop 2	50	29.36	2.86 ± 0.62 (*n* = 9)	0.921 ± 0.03 (*n* = 35)
Pop 3	37	9.56	2.01 ± 0.24 (*n* = 9)	0.907 ± 0.33 (*n* = 22)
Box turtles				
Pop 1	27	174.84	8.56 (*n* = 1)	0.791 ± 0.24 (*n* = 11)
Pop 2	89	514.37	8.68 ± 1.45 (*n* = 11)	0.729 ± 0.22 (*n* = 38)
Pop 4	212	930.20	7.12 ± 1.49 (*n* = 40)	0.686 ± 0.76 (*n* = 50)

### 3.2 Genetic Diversity and Inbreeding Coefficient

Measures of genetic diversity and inbreeding were similar across the three study populations within each species (Table 2). Mean allelic richness was 5.8–7.0 over all loci in the spotted turtle study populations, and 6.3–14.3 in the box turtle study populations, with a mean of 0.4–0.6 private alleles per locus in spotted turtles and 0.2–1.2 private alleles per locus in box turtles (Table 2). Mean number of rarefied alleles in each population was similar to mean allelic richness in the spotted turtle populations, but in the box turtle populations mean number of rarefied alleles ranged from 4.0 to 4.5 (Table 2). Spotted turtle study populations had observed and expected heterozygosities of 0.72–0.78 and 0.73–0.79, respectively (Table 2). In the eastern box turtle study populations, expected heterozygosities ranged from 0.81 to 0.88 while observed heterozygosities were 0.63–0.64 (Table 2). Finally, the inbreeding coefficients from the three spotted turtle study populations were 0.01–0.03, whereas for the three box turtle study populations inbreeding coefficients were 0.25–0.29 (Table 2).

**TABLE 2:** Mean allelic richness, number of private alleles, rarefied allelic richness, observed heterozygosity (*H*_o_), expected heterozygosity (*H*_e_), and inbreeding coefficient (*F*_is_) in three study populations of spotted turtles ( *Clemmys guttata* ) and eastern box turtles ( *Terrapene carolina carolina* ) in Lucas County, Ohio (Pops 1 and 2), and Barry (Pop 3) and Calhoun Counties (Pop 4), Michigan, USA in 2018 and 2019.

	Allelic richness	Private alleles	Rarefied allelic richness	*H*_o_	*H*_e_	*F*_is_
Spotted turtles						
Pop 1 (*n* = 14)	6.88 ± 2.75	0.38 ± 0.74	6.50 ± 2.61	0.75 ± 0.18	0.77 ± 0.12	0.03 ± 0.17
Pop 2 (*n* = 15)	7.00 ± 2.33	0.63 ± 0.74	6.40 ± 2.00	0.78 ± 0.20	0.79 ± 0.08	0.02 ± 0.17
Pop 3 (*n* = 11)	5.75 ± 2.05	0.50 ± 1.07	5.88 ± 2.03	0.72 ± 0.23	0.73 ± 0.23	0.01 ± 0.10
Box turtles						
Pop 1 (*n* = 8)	6.33 ± 2.34	0.17 ± 0.41	3.99 ± 0.75	0.64 ± 0.24	0.81 ± 0.24	0.25 ± 0.28
Pop 2 (*n* = 37)	13.50 ± 5.01	0.83 ± 0.98	4.53 ± 0.41	0.63 ± 0.24	0.88 ± 0.04	0.29 ± 0.26
Pop 4 (*n* = 44)	14.33 ± 3.78	1.17 ± 0.75	4.49 ± 0.41	0.63 ± 0.19	0.87 ± 0.04	0.27 ± 0.21

*Note:* All values are shown as means over eight (spotted turtle) or six (box turtle) microsatellite loci ± SD.

### 3.3 Egg‐Hatching Success and Hatchling Survival to Overwintering

In comparing metrics of reproductive success as potential evidence of inbreeding depression between spotted turtles and eastern box turtles, we pooled egg‐hatching success and hatchling survival to overwintering across the three study populations for each species. Overall, egg‐hatching success (excluding predation) was 80% in spotted turtles and 58% in eastern box turtles (*t* = 4.33, *p* < 0.0001; Table 3). Our previous research in the same study populations documented nest predation rates of 6% for spotted turtles and 58% for eastern box turtles (Refsnider et al. 2022); thus, box turtles experience substantially higher rates of both nest predation and mortality of eggs from intact nests compared to spotted turtles. Hatchling survival to overwintering was also higher in spotted turtles compared to eastern box turtles. Our conservative estimates of hatchling survival to overwintering, using only hatchlings whose fates were known with certainty, were 95% for spotted turtles and 71% for eastern box turtles (*t* = 2.67, *p* = 0.01; Table 3). When we included additional hatchlings whose fates were presumed but not known with certainty, we estimated that 82% of spotted turtle hatchlings survived to overwintering and 59% of eastern box turtle hatchlings survived to overwintering (*t* = 2.63, *p* = 0.01; Table 3).

**TABLE 3:** Per cent of eggs that hatched (egg‐hatching success) and per cent of hatchlings that survived to overwintering (hatchling survival rate) pooled over three study populations of spotted turtles ( *Clemmys guttata* ) and eastern box turtles ( *Terrapene carolina carolina* ) in Lucas County, Ohio, and Barry and Calhoun Counties, Michigan, USA in 2018 and 2019.

	Egg‐hatching success	Hatchling survival rate^a^
Spotted turtles	79.7% (*n* = 34 nests)	Known: 19/20 = 95% Presumed: 28/34 = 82%
Box turtles	58.1% (*n* = 68 nests)	Known: 28/39 = 71% Presumed: 40/68 = 59%

*Note:* The dataset and estimates of egg‐hatching success and hatchling survival to overwintering for both species have been previously published (Refsnider et al. 2022; dataset available in Refsnider, Carter, et al. 2021, “SWGHatchlings.csv”).

^a^ “Known” rates were calculated including only hatchlings whose fates were known with certainty. “Presumed” rates were calculated based on observed loss of transmitter signal relative to expected transmitted battery expiration and date (see Section 2).

## 4 Discussion

Inbreeding in small populations is a serious concern for conservation biologists because of its potential to lead to a decline in reproductive fitness due to inbreeding depression (e.g., Crnokrak and Roff 1999; Heber and Briskie 2010). Across a wide range of taxa, a higher inbreeding coefficient is correlated with higher inbreeding depression (Vega‐Trejo et al. 2022), which demonstrates the potential for high rates of inbreeding to contribute to population extirpation. Importantly, the apparent absence of inbreeding depression in a population does not mean there are no inbreeding effects present (Hedrick and Kalinowski 2000); thus, effects of inbreeding are likely underestimated in wild populations where detailed pedigrees and data on individuals' lifetime reproductive success are usually lacking (Keller and Waller 2002; Hedrick and Garcia‐Dorado 2016). At our study sites, spotted turtles had smaller population sizes, occupied smaller geographic areas, had smaller home ranges, and had higher baseline physiological stress levels compared to eastern box turtle populations. These characteristics might be expected to predispose the spotted turtle populations to higher rates of inbreeding and potentially to inbreeding depression. Instead, we found that the eastern box turtle populations had much higher inbreeding coefficients than the spotted turtle populations. Moreover, lower rates of egg‐hatching success and hatchling survival to overwintering in the eastern box turtle compared to the spotted turtle populations suggest that the box turtle populations may be experiencing inbreeding depression, whereas inbreeding depression was not immediately evident in the spotted turtle populations, all of which demonstrated high rates of both egg‐hatching success and hatchling survival to overwintering.

In our study, we calculated population‐level coefficients of inbreeding (*F*_is_), rather than using pedigrees to determine the degree of inbreeding of specific individuals. Thus, we cannot directly correlate individuals' degree of inbreeding with their reproductive success, as has been done in other studies (e.g., Vega‐Trejo et al. 2022). Nevertheless, at the population level, we found that populations with higher coefficients of inbreeding also had lower metrics of reproductive success, which suggests that such populations may be experiencing inbreeding depression. It is important to acknowledge that, even when inbreeding occurs, it does not necessarily result in easily detectable inbreeding depression. Our calculation of the inbreeding coefficient *F*_is_ only measures inbreeding relative to what would be expected under random mating; thus, inbreeding in the spotted turtle populations might be detectable if it were measured as, for example, the probability of two individuals being identical by descent, or if inbreeding occurs within subdivided, isolated populations (Keller and Waller 2002). Additionally, tradeoffs between fitness components at different life stages may exist such that inbreeding depression may not be evident at all life stages (Armbruster and Reed 2005). Thus, while inbreeding depression may not be evident in spotted turtles at the early life stages we measured here, it may become evident in later life stages or even subsequent generations (Pusey and Wolf 1996; García‐Fernández et al. 2012). Importantly, however, the impacts of inbreeding depression are likely greatest in earlier life stages (Hedrick and Garcia‐Dorado 2016), such that less inbred individuals are more likely to survive past the juvenile stage than are more inbred individuals (Duntsch et al. 2023). Our three eastern box turtle study populations exhibited high rates of inbreeding and lower metrics of both egg‐hatching success and hatchling survival compared to spotted turtles at the same study sites, which strongly indicates inbreeding depression operating in the box turtle populations. Why might box turtles, but apparently not spotted turtles, be exhibiting potentially deleterious levels of inbreeding?

In birds and mammals, sex‐biased dispersal is a crucial mechanism for inbreeding avoidance (e.g., Pusey 1987; Galezo et al. 2022). Much less is known about patterns of sex‐biased dispersal in turtles, but a population of eastern box turtles at the northern extent of the species' geographic range exhibited male‐biased dispersal (Moore et al. 2020), whereas no sex‐biased dispersal was evident in spotted turtles even when individuals had high relatedness over small spatial scales (Liebgold et al. 2023). Thus, spotted turtles may rely on kin recognition, rather than dispersal by one sex, to avoid mating with close relatives (reviewed in Pusey and Wolf 1996). Kin recognition is likely to be particularly important in reptiles such as spotted turtles, which often hibernate communally and mate upon spring emergence (Litzgus et al. 1999). In contrast, eastern box turtles tend to hibernate individually and mate opportunistically throughout the year (Willey and Sievert 2012), which could increase the likelihood of random mating in this species. Eastern box turtles have larger home ranges than spotted turtles in our study populations, as well as others across both species' ranges (Buchanan et al. 2017; Habeck et al. 2019; Roe et al. 2020; O'Dell et al. 2021). Moreover, the total patch size occupied at each of our study sites was considerably larger for eastern box turtles than for spotted turtles. If box turtles require larger areas than spotted turtles to procure resources including nesting areas, overwintering sites, and mates, then box turtles may be more vulnerable than spotted turtles to fragmentation of habitat. In particular, if eastern box turtles rely on male‐biased dispersal to avoid inbreeding, but are prevented from dispersing by barriers such as roads or inhospitable habitat, sex‐biased dispersal will likely fail to prevent inbreeding. Dispersal barriers could, in turn, lead to high rates of inbreeding and lower reproductive success, which is consistent with our observations and could be indicative of inbreeding depression in our study populations. Further research is needed to ascertain the degree to which either spotted turtles or eastern box turtles recognize close kin, and whether kin recognition functions to minimize inbreeding in either species. Similarly, research is needed to determine whether habitat fragmentation limits dispersal of eastern box turtles and contributes to higher rates of inbreeding in populations in more fragmented landscapes.

While inbreeding depression has been hypothesized to increase with environmental stress, experimental evidence supporting this prediction has been mixed (e.g., Armbruster and Reed 2005; Marr et al. 2006; Kristensen et al. 2008; Waller et al. 2008; Fox and Reed 2010; García‐Fernández et al. 2012; Vega‐Trejo et al. 2022). A general trend may be that inbreeding increases individuals' susceptibility to environmentally inflicted stress or mortality (Keller and Waller 2002; Willi et al. 2006); for example, in flour beetles, environmental conditions that were mildly stressful to outbred individuals were perceived as highly stressful by inbred individuals (Fox and Reed 2010). In our study populations, habitat fragmentation by roads and trails, and the human activity associated with them, is a likely environmental stressor, which could both promote inbreeding by preventing individuals from dispersing to mate with unrelated conspecifics and potentially increase the mortality risk of inbred and therefore less fit individuals.

While inbreeding depression in the spotted turtle populations may be evident at different life stages than those examined here, it is also possible that past genetic purging removed the most deleterious alleles from the populations (e.g., Hedrick and Garcia‐Dorado 2016), or that the long generation time of spotted turtles means that the inbreeding coefficients measured here are more reflective of the dynamics of historical, larger populations rather than contemporary, more isolated populations (Keller and Waller 2002; Marsack and Swanson 2009; Davy and Murphy 2014). Regardless, genetic diversity, inbreeding prevalence, and metrics of reproductive output should continue to be monitored in populations of both species so management actions can be taken if population genetic health declines. Comparing allelic richness within the juvenile cohort to that of the adults measured here would also be useful for ascertaining whether genetic diversity is declining in younger generations (Clark et al. 2024). The high frequency of inbreeding we detected in the box turtle study populations, in combination with decreased reproductive success compared to the spotted turtle populations, is cause for concern for the eastern box turtle populations in the North American Great Lakes oak savanna landscape. It seems likely that habitat loss and fragmentation in this region has contributed to inbreeding effects in this relatively vagile species, for example by obstructing dispersal that would otherwise promote mating among unrelated conspecifics. We suggest that a top priority for conserving these vulnerable turtle species should be the preservation of intact tracts of habitat, restoring connectivity among sub‐populations where possible, and preventing further fragmentation of occupied habitat by roads to promote gene flow and random mating.

## Author Contributions

**Paige K. Madden:** formal analysis (equal), investigation (equal), writing – review and editing (equal). **Sarah E. Carter:** formal analysis (equal), investigation (equal), writing – review and editing (equal). **Austin C. Hulbert:** formal analysis (equal), investigation (equal), writing – review and editing (equal). **Henry M. Streby:** conceptualization (equal), formal analysis (equal), funding acquisition (equal), investigation (equal), project administration (equal), writing – review and editing (equal). **Jeanine M. Refsnider:** conceptualization (equal), data curation (equal), formal analysis (equal), funding acquisition (equal), investigation (equal), project administration (equal), writing – original draft (equal).

## Ethics Statement

All animals were handled in accordance with all required state and local scientific research permits, and with the University of Toledo's Institutional Animal Care and Use Committee (protocol #108797).

## Conflicts of Interest

The authors declare no conflicts of interest.

## Data Availability

Data are deposited in Dryad: https://doi.org/10.5061/dryad.pvmcvdnwc (Refsnider et al. 2025).
